# Effectiveness, enjoyment, and meaningfulness of a virtual reality gait-based fall prevention exergame in community-dwelling healthy older adults: an interdisciplinary pilot study

**DOI:** 10.3389/fpsyg.2025.1610377

**Published:** 2025-06-03

**Authors:** Celina Ciemer, Nadja Schott, Thomas Jürgen Klotzbier, Sabiha Ghellal

**Affiliations:** ^1^Institute for Sport and Movement Science, University of Stuttgart, Stuttgart, Germany; ^2^Institute for Games, Stuttgart Media University, Stuttgart, Germany

**Keywords:** virtual reality, fall prevention, user experience, game design, human movement science, exergaming, interdisciplinary research, accidental falls

## Abstract

**Introduction:**

Falls are a prevalent health concern among older adults, potentially resulting in substantial physical, psychological, and social ramifications. Interventions aimed at fall prevention require effectiveness, enjoyment, and meaningfulness (EEM). As gait impairments are a key factor in fall risk, integrating natural locomotion and cognitive skills through single- and dual-task training is essential. We developed *EXploVR*, a fully immersive virtual reality exergame that integrates natural gait and promotes EEM. This interdisciplinary pilot study examined the EEM of *EXploVR* in healthy, community-dwelling older adults.

**Methods:**

Forty-six participants were assigned to an intervention or passive control group using a single-blinded, quasi-randomized design. Over three weeks, the intervention group completed two 60-min sessions weekly. Baseline, mid-, and post-assessments included single- and dual-task gait (instrumented normal and tandem walks, counting task), lower limb strength and transitional movement (instrumented Five Times Sit-to-Stand test, 5xSTS), and static postural control (instrumented sway tests). In-game performance (time-to-complete) was recorded. Enjoyment was assessed via the Flow Short Scale (FKS), Physical Activity Enjoyment Scale (PACES-S) and adaptations, and Exergame Enjoyment Questionnaire (EEQ). Meaningfulness was assessed via the Activities-specific Balance Confidence Scales (ABC-6, ABC-8) and custom questions on perceived safety, fear of falling, daily-life integration, emotional challenges, and perceived effectiveness for fall prevention.

**Results:**

Data from 32 participants (16 intervention, age = 70.00 ± 3.33 years; 16 control, age = 68.38 ± 5.54 years) were analyzed. Significant improvements were found in walking gait speed (*p* = 0.019) and tandem gait speed (*p* = 0.032). Under dual-task conditions, only tandem gait speed improved significantly (*p* = 0.022). 5xSTS showed a significant interaction for total duration (*p* = 0.023), while postural sway demonstrated non-significant improvement trends. In-game station completion time improved significantly in 5 of 6 sets (*p* < 0.05). Enjoyment remained high or increased, and meaningfulness was supported by positive trends in ABC-6 (*p* = 0.094) and significant gains for ABC-8 (*p* = 0.026). Custom questions further supported these findings.

**Conclusion:**

This study suggests that EXploVR is effective and enjoyable while fostering meaningfulness. Further research with larger samples and extended interventions is needed to confirm long-term effects and daily-life transfer.

## 1 Introduction

In its World Report on Aging and Health (World Health Organization, 2015), the World Health Organization (WHO) defines the development and maintenance of functional abilities as “healthy aging” and posits that this is the foundation of well-being in old age. According to the WHO (Rudnicka et al., 2020), functional ability encompasses an individual's capacity to meet basic needs, make independent decisions, remain mobile, establish and maintain social relationships, and actively participate in society. Thus, motor and cognitive performance represent a pivotal factor in quality of life, defined as a person's actual or potential ability to perform tasks or activities, and correlates with mental and physical health.

A decline in motor and cognitive performance, accompanied by a simultaneous decline in physical and sporting activities, is associated with a significantly increased risk of falls. More than 30% of older adults experience a fall each year (Rapp et al., 2014; Rubenstein, 2006). Approximately 40% to 60% of individuals who experience a fall sustain injuries, with 10% of these cases resulting in serious injuries, including fractures or head trauma (Kenny et al., 2017). Risk factors include mobility and sensory factors such as walking speed, lifestyle-related factors such as physical activity levels, and psychological and medical factors such as cognitive impairment (Ek et al., 2018). Even without physical harm, falls can lead to a decline in self-confidence and self-esteem, reduced activity levels, and social isolation. Such effects negatively impact wellbeing and quality of life and may accelerate physical decline and the aging process (Close et al., 1999; Painter et al., 2012; Schott, 2007).

Most falls occur during locomotion (Talbot et al., 2005), highlighting that gait impairments are a key cause of falls. Research indicates that gait impairments and falls are not only related to limitations in static and dynamic postural control from age-related declines in muscle strength, endurance, and performance (Kamińska et al., 2015) but are also influenced by cognitive impairments (Montero-Odasso et al., 2012) and difficulties with dual-task activities (Beauchet et al., 2009; Springer et al., 2006), such as walking while talking. The use of cognitive resources to accomplish dual- or multi-tasks appears to result in a trade-off between postural control and the ability to divide attention between different tasks. However, dual-task training reduces fall incidence in older adults by shifting attention based on the difficulty and priority of a task from the motor to the cognitive task (Gallou-Guyot et al., 2020b; Khan et al., 2022; Varela-Vásquez et al., 2020). The World Falls Guidelines Task Force (Montero-Odasso et al., 2022) recommends that individuals who have experienced a fall in the past year, those with a fear of falling, and older individuals with a walking speed below 0.8 m/s undergo regular screening and interventions. In addition, a clear link between gait speed and falls is discovered by showing a protective effect against falling for people with a higher gait speed (Smith et al., 2016). The meta-analysis of Sherrington et al. (2017) demonstrated that exercise-based interventions could reduce fall rates by more than 20%. The effects were most pronounced when balance training was involved and training was conducted for a total of more than three hours per week (39% reductions in the fall rate) focusing on (1) standing exercises with a gradually unstable support surface (e.g., two-legged stance, two-legged stance, two-legged stance), (2) dynamic and reactive movements (e.g., tandem gait), (3) the use of posturally relevant muscle groups (e.g., heel stand, toe stand), and (4) variations in sensory input (e.g., standing with eyes closed).

Traditional fall prevention programs focusing only on functional capacities have demonstrated limitations, such as low adherence and motivation among older adults (Choi et al., 2017; Merom et al., 2012; Nyman and Victor, 2011). Therefore, incorporating psychological and emotional factors into the training design is crucial to addressing the needs and interests of older adults. These factors are essential for fostering enjoyable experiences and creating meaning that extends into daily life, such as enhancing fall-related self-efficacy and confidence (Chao et al., 2015; Ge et al., 2022; Gerling et al., 2012; Mueller et al., 2020; Retz et al., 2023; Ringgenberg et al., 2022; Testa and Simonson, 1996). Consequently, the interplay between Effectiveness, Enjoyment, and Meaningfulness (EEM) becomes paramount (Retz et al., 2024, 2023).

Exergames offer the inherent potential to combine effective motor-cognitive training with enjoyment and meaningfulness in a single application, primarily due to their interdisciplinary nature, which fuses exertion and games (Kappen et al., 2019; Martin-Niedecken et al., 2019; Sinclair et al., 2009). They allow players to interact physically, using body movements or specific body parts to engage with sensor-based systems (Oh and Yang, 2010; Röglin et al., 2023; Subramanian et al., 2020). The effectiveness of exergames stems from their design, which includes dual tasks that challenge both motor and cognitive skills (Martin-Niedecken et al., 2019; Oh and Yang, 2010; Wang et al., 2021). Enjoyable and positive multi-dimensional experiences can be established by the use of game design methods (Abeele et al., 2020; Belchior et al., 2012; Isbister, 2016; Kappen et al., 2019; Lyons, 2015; Marston, 2013; Martin-Niedecken et al., 2020). At the same time, meaningfulness can be created through the design of the training and the user experience by addressing the psychological needs and emotions of the target audience (Chao et al., 2015; Ge et al., 2022; Gerling et al., 2012; Retz et al., 2023; Ringgenberg et al., 2022).

Systematic reviews demonstrate that exergames have the potential to support fall prevention by addressing three key aspects. First, they have been shown to improve motor and cognitive abilities related to fall risk. This includes enhancements in static and dynamic postural control (Chen Y. et al., 2021; Donath et al., 2016; Fang et al., 2020; Neri et al., 2017; Piech and Czernicki, 2021), dual-task performance (Gallou-Guyot et al., 2020a; Ogawa et al., 2016; Schoene et al., 2014), cognitive functions such as attention and executive control (Choi et al., 2017; Ogawa et al., 2016; Schoene et al., 2014), and overall fall risk reduction (Alhagbani and Williams, 2021; Lapierre et al., 2023). Second, reviews indicate that exergames can enhance perceived enjoyment by focusing on usability and acceptance (Buyle et al., 2022; Nawaz et al., 2016). Third, they can be meaningful by reducing the fear of falling and enhancing confidence (Ge et al., 2022; Neri et al., 2017; Schoene et al., 2014). However, exergame research often adopts a narrow, discipline-specific perspective, focusing solely on either functional or psychological factors and treating the development and evaluation of exergames as separate processes. To fully realize the potential of fall prevention exergames, adopting an approach that aligns exergame design with study design to enable a clear understanding of the rationale behind outcomes and ensure reproducibility, transparency, and further refinements is essential (Ciemer et al., 2025).

A considerable body of research underutilizes the vast possibilities offered by digital technologies. The reliance on pre-existing systems such as the Nintendo Wii or Xbox 360, along with solutions that limit movement to confined spaces like Kinect sensors and screen-based setups, has shaped many approaches. These typically involve standing in different positions such as parallel, semi-tandem, or tandem stances, functional reach, weight shifting, stepping in place, or stepping in various directions (Ciemer et al., 2025). However, this approach fails to acknowledge the significance of locomotion. The vast space of possibilities of fully immersive virtual reality (VR) offers a promising technological enabler for exergame interventions. VR enables complete immersion in virtual worlds, creating a high level of presence. It holds the potential to create rich experiences that allow for natural physical movements and locomotion techniques in the real world to interact with digital elements (Campo-Prieto et al., 2021; Jerald, 2015; Jung et al., 2009; Patel et al., 2006; Slater et al., 2009).

In order to intentionally address the objectives of the EEM triad, it is imperative to use interdisciplinary design models and procedures, such as the Co-Creative Interdisciplinary Exergame Design Process Model with Extended Reflection (CIEMER) (Retz et al., 2024). These interdisciplinary frameworks provide a comprehensive perspective on the entire design and evaluation process encompassing objectives, design and development, the exergame intervention itself, and evaluation (Ciemer et al., 2025). By applying CIEMER, *EXploVR: A Mountain Mastery* was created as a fully immersive VR platformer exergame for fall prevention (Retz et al., 2023). This exergame emphasizes effectiveness, enjoyment, and meaningfulness, allowing natural physical movements and gait techniques to interact within the virtual environment. EXploVR is designed to integrate motor and cognitive training in single- and dual-task conditions, thereby achieving effectiveness. The exergame is designed to promote enjoyment by appealing to Achiever and Free Spirit user types and their needs for competence, autonomy, and safety during training. Furthermore, EXploVR aims to create meaningful experiences by addressing common fears, such as falling and heights, while enhancing balance confidence and promoting advanced activities of daily living. These activities are essential for community-based leisure pursuits, such as hiking.

This interdisciplinary pilot study aims to explore the effectiveness, enjoyment, and meaningfulness of EXploVR in community-dwelling healthy older adults. Specifically, it examined whether EXploVR could:

1. improve motor and cognitive parameters (single- and dual-task gait, lower limb strength, and transitional movement ability as well as static postural control),
2. increase enjoyment during the intervention (flow experience and exergame enjoyment), and
3. create meaningfulness during training (safety, fear of falling, and perceived effectiveness) and extend into daily life (balance confidence, integration into daily life, and importance of emotional challenges).

## 2 Materials and methods

### 2.1 Study design and participants

Using an experimental design, an interdisciplinary pilot study was structured as a two-arm, single-blinded, quasi-randomized controlled trial with an active intervention and a passive control group (1:1 allocation ratio). Participants were assigned quasi-randomly based on the enrollment order and were blinded to their group allocation. The methods and results from the EXploVR quasi-randomized controlled trial were reported in accordance with the Consolidated Standards of Reporting Trials (CONSORT) (Hopewell et al., 2025).

We recruited community-dwelling healthy older adults from Stuttgart (Germany) and nearby areas between June and September 2024 by mostly visiting different senior sports clubs, distributing flyers and posters, publishing newsletter articles, and word of mouth.

Following an initial telephone screening based on Warburton et al. (2021), eligible individuals were invited to participate if they met the inclusion criteria, which required participants to be healthy, community-dwelling older adults aged 60 to 75, independent in daily living activities and living arrangements, able to walk more than 400 meters without a walking aid, rise from a chair without hand support, walk a straight line and navigate crowded spaces without difficulty. Participants also needed to be willing and able to give informed consent. Exclusion criteria included any health conditions affecting gait or physical function testing, unstable or acute medical conditions precluding exercise, conditions causing muscle wasting (e.g., secondary sarcopenia), cognitive impairments (e.g., dementia or mild cognitive impairment), recent heart attack (within the past six months), severe cardiovascular disease (e.g., stroke), uncontrolled hypertension (greater than 160/100 mmHg), degenerative diseases (e.g., Parkinson's disease), other acute or chronic medical or psychiatric conditions, blindness or severe visual impairment, or insufficient German language skills. Those eligible were asked to provide informed written consent.

Sample size estimation was computed with G*Power 3.1 software (Heinrich Heine Universität Düsseldorf) (Faul et al., 2009). As a statistical test, the repeated-measures ANOVAs within-between interactions have been considered. Considering the findings of previous meta-analyses, which indicated small to moderate effects of exergaming on physical and cognitive functions in older adults, the effect size was calculated to be 0.25 (Hai et al., 2022; Jiang et al., 2022). The significance level (α) was set at 0.05, and the power (1β error probability) at 0.80. We have 2 separate groups and 3 measurements. 14 participants per group were required. We expected a 30% to 40% dropout rate to ensure the study's statistical power. As a result, we aimed to recruit 20 participants per group; thus, in total, 40 participants.

The study was performed in line with the principles of the Declaration of Helsinki. The ethics committee of the University of Stuttgart, Germany has approved the study protocol (AZ. 23-052).

### 2.2 EXploVR exergame

#### 2.2.1 Exergame development

Following the proposal outlined in CIEMER (Retz et al., 2024), the EEM triad was employed to define the objectives of the exergame, with these objectives being addressed through a collaborative effort involving expert groups from the domains of Human Movement Science, Experience Design, and Game Design, as well as representatives of the target group of community-dwelling healthy older adults, who served as experts of their own needs (Retz et al., 2023). The development process adhered to established concepts and procedures from the involved disciplines to facilitate a clear understanding of rationales and ensure reproducibility.

The interdisciplinary exergame EXploVR features 36 exercise stations grouped into six station sets. Each set includes three stations, designed with two levels of difficulty. EXploVR is divided into two levels, each containing three station sets. The main objective within the exergame is to navigate various mountain routes and locate a portal leading to following routes to reach the mountain's summit, enjoy the hiking environment and physical activity, and overcome personal limits and fears.

**Figure 1:**
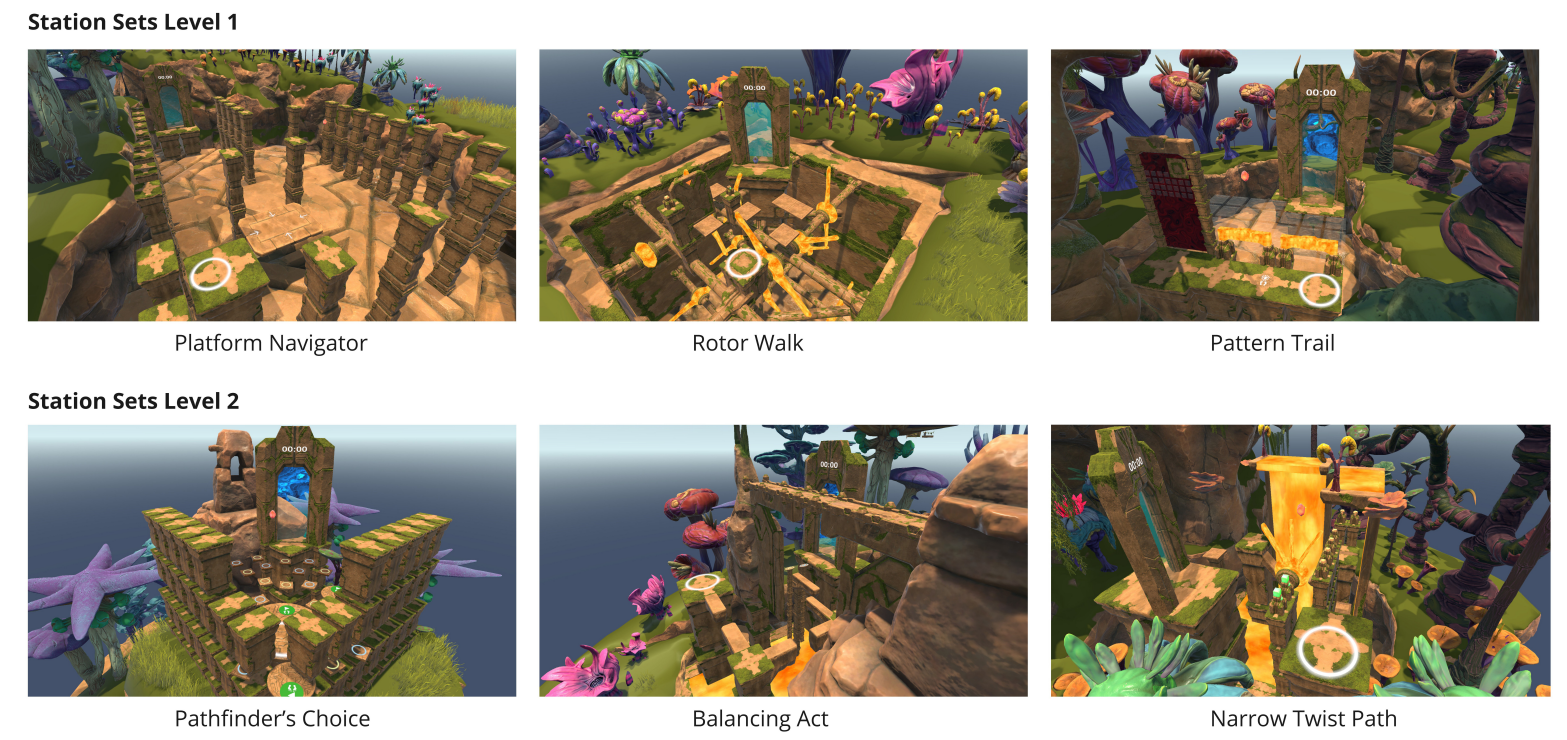
Level and station sets with exemplary stations of EXploVR: A Mountain Mastery.

**Figure 2:**
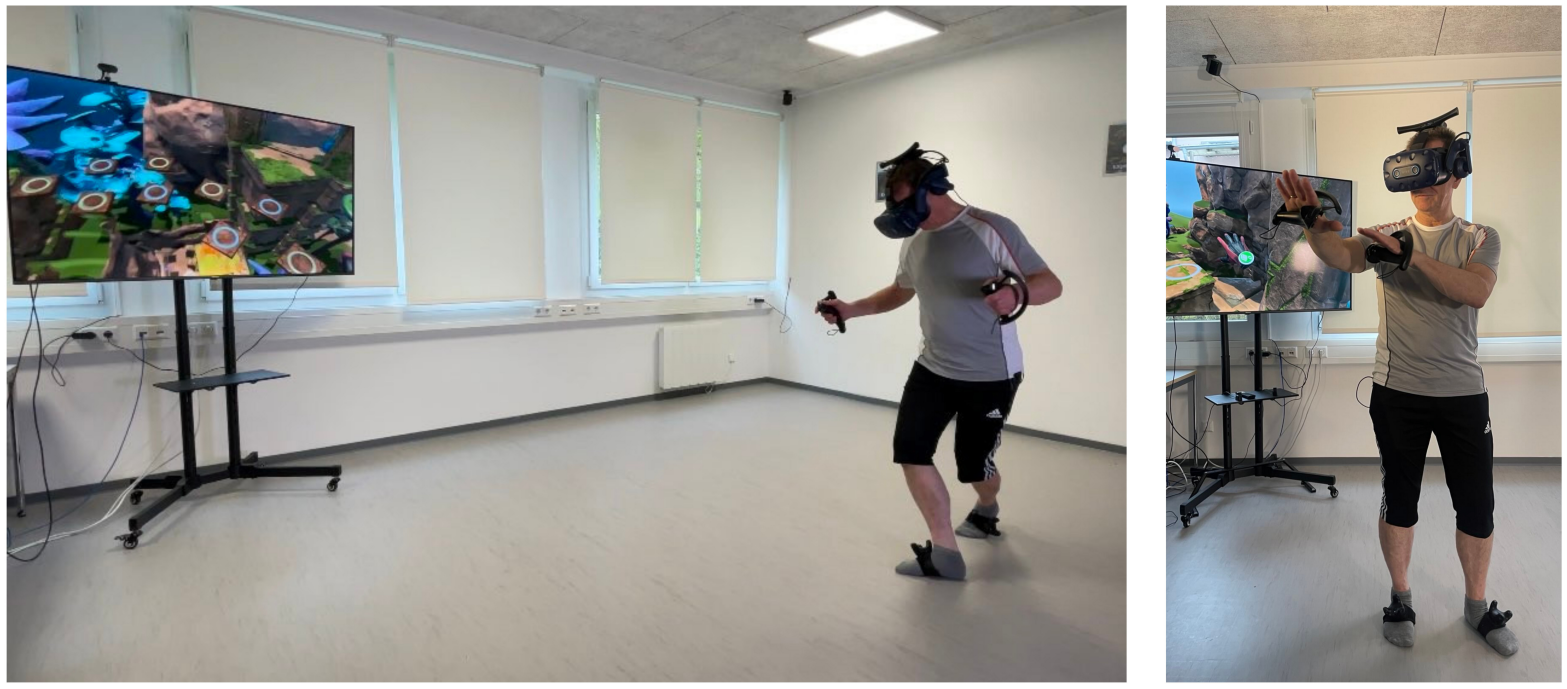
Participant during EXploVR training in a tandem variation walk with turns and a cognitive task.

In the category of effectiveness, each station set is based on multi-faceted motor-cognitive training. The selection of fall prevention exercises follows the categorization proposed by Ciemer et al. (2025), which divides postural control into three components: static postural control (standing), dynamic postural control (gait), and dynamic postural control (mobility). This categorization also extends to lower limb strength and cognitive functions. Gentile's (2000) taxonomy was applied by grouping different types of motor tasks to structure tasks with increasing difficulty. Gentile's taxonomy is a theoretical framework that facilitates the development of skills, comprising two primary perspectives: the environmental context and the action function. The performance conditions can be constant (absence of intertrial variability) or variable (presence of intertrial variability) across trials. The integration of action function, encompassing body stability or body movement in conjunction with object manipulation, or without object manipulation, in conjunction with the environmental context, gives rise to a coherent sequence and progression of skills. A task-based exercise approach was implemented, incorporating both single-task and predominantly dual-task exercises. The specific foci of the station sets are detailed in Table 1, with example stations of the sets shown in Figure 1 and a participant during training is presented in Figure 2.

**Table 1:** Overview of motor and cognitive focus for different station sets.

**Station set**	**Main focus: motor**	**Main focus: cognition**
**Level 1**		
Platform Navigator	Dynamic postural control: mobility (e.g., stepping in different directions, functional reach, weight shifting). Lower limb strength (e.g., squat and lunge variations).	Executive functions (e.g., planning, decision-making, inhibition).
Rotor Walk	Dynamic postural control: gait (e.g., normal walking, walking while avoiding obstacles, walking with turns).	Complex attention (e.g., selective attention, divided attention, processing speed).
Pattern Trail	Dynamic postural control: gait (e.g., normal walking, walking with precision, walking with turns).	Executive functions (e.g., working memory, planning).
**Level 2**		
Pathfinder's Choice	Dynamic postural control: gait (e.g., walking with precision, semi-tandem and tandem walk variations, walking with turns). Static postural control (e.g., one leg stand).	Executive functions (e.g., decision making, planning).
Balancing Act	Dynamic postural control: gait (e.g., walking with precision, semi-tandem and tandem walk variations, walking with turns). Static postural control (e.g., one leg stand).	Complex attention (e.g., selective attention, processing speed).
Narrow Twist Path	Dynamic postural control: mobility (e.g., sidestepping, cross-stepping, body rotation). Lower limb strength (e.g., squat variations).	Executive functions (e.g., planning, decision making, cognitive flexibility, responding to feedback, inhibition).

The objectives within the enjoyment category were derived from the Hexad User Types (Altmeyer et al., 2019; Tondello et al., 2017, 2016), specifically targeting the Achiever and Free Spirit types. Their needs for competence and autonomy were addressed within the exergame in the context of the platformer genre (Retz et al., 2023). Core platformer elements were integrated to achieve this, including movement between platforms, progression through trial and error, and challenges involving obstacles, movement aids, triggers, and collectible items (Melcer and Cuerdo, 2020; Smith et al., 2008). Additionally, features were designed to cater specifically to the targeted user types. These included multiple self-contained challenges, visible progression, opportunities for exploration within the virtual world, and non-linear gameplay across different difficulty levels.

In the meaningfulness category, EXploVR aims to fulfill the need for safety during training by fostering a sense of security and building confidence in performing exergame activities. Beyond training, it is designed to support and sustain community participation by maintaining Advanced Activities of Daily Living (Briede-Westermeyer et al., 2023; Czaja et al., 2019; De Vriendt et al., 2012), in enabling routine leisure activities such as hiking within a community. The exergame fosters social engagement and safety, particularly by alleviating fear of falling and improving movement in challenging outdoor environments, such as navigating diverse hiking terrains (Retz et al., 2023). These needs are supported through features such as practicing virtual missteps, providing visual safety cues and control options, confronting the fear of falling in outdoor environments at different heights, and encouraging participation in challenging real-world scenarios.

#### 2.2.2 Exergame levels and station sets

In *level 1* of EXploVR, the station set *Platform Navigator* is designed to navigate a moving platform through a dynamic parkour using both hand-operated and foot-operated controls. Successfully navigating the course requires the player to switch between these two control types to overcome obstacles and reach the portal. The control layouts exhibit a high degree of variability, with directional inputs either aligning naturally or being placed in unconventional ways. In the subsequent station set, titled *Rotor Walk*, participants are tasked with navigating a course comprising stationary and rotating rotors at varying heights. This requires precise timing to successfully traverse the rotors while crossing platforms of different sizes and gaps. As the challenge progresses, it introduces overlapping rotors, and participants must retrieve and precisely place a cube to unlock a door. In the subsequent set, titled *Pattern Trail*, participants are tasked with memorizing platform patterns, applying these patterns to unlock the path, and traversing the path to the portal. The path is revealed by floor buttons marked with an eye icon. As the challenge progresses, the patterns become increasingly complex, and additional obstacles, such as barriers and moving hazards, are introduced.

In *level 2*, the first station set is *Pathfinder's Choice*, which involves navigating through various challenges en route to a portal. Routes must be selected, stepping platforms must be chosen, and movements must be executed precisely to progress. The pathways feature a range of platform sizes, gaps, and configurations, necessitating strategic decision-making and precise foot placement. Single-leg stances must operate foot-activated switches, and rotating elements must be halted at the opportune moment. In the subsequent set, titled *Balancing Act*, the participant must traverse narrow beams and foot-shaped platforms while navigating a path to the portal. This environment is designed to evoke a state of emotional distress, necessitating the use of balance, precision, and strategic foot placement. The beams gradually narrow, incorporating gaps and timed gates, which require the adept use of various walking techniques, including semi-tandem and tandem steps, as well as steps with the dominant and non-dominant foot. The foot-shaped platforms, designated for specific feet, must be used following the designated foot switches, which are positioned at knee height and require activation to raise the platforms. In the subsequent station set, titled *Narrow Twist Path*, navigating through the path while managing obstacles and cube placement necessitates the execution of side-stepping, cross-stepping, and precise body rotations. Platforms and beams require 180-degree rotations to circumvent lava walls and ceilings, necessitating squat-like positions to ensure safe progression. Cubes must be collected and strategically positioned on pillars to elevate platforms and avert entrapment.

### 2.3 Procedure

The study was conducted in a university laboratory with an open area measuring 5 by 9 meters. This allowed for installing a 4-by-4-meter VR training field, the necessary technical equipment, chairs for breaks, and an area including a walking corridor for movement tests. The VR setup utilized the HTC Vive Pro 2 system in a wireless configuration. Additionally, participants wore HTC Vive Trackers on their feet and used Valve Index Controllers with their hands. The training area had four VIVE base stations mounted on the ceiling, ensuring precise positioning and movement detection. The virtual environment featured a guardian system that displayed the boundaries of the training area, enabling participants to always perceive the limits of the space. Despite the virtual nature of the obstacles and challenges, two trained supervisors were present to provide physical assistance as needed. A large external screen displayed a live feed of the participants' VR view, facilitating supervision.

The study lasted three months. The intervention group participated in six training sessions, scheduled individually and administered over three weeks. Each session was 60 min, with breaks of at least 2 days and no more than 4 days between sessions. Participants could take breaks at any time during the sessions and remove the glasses if needed. The control group continued their habitual activities throughout the study.

**Figure 3:**
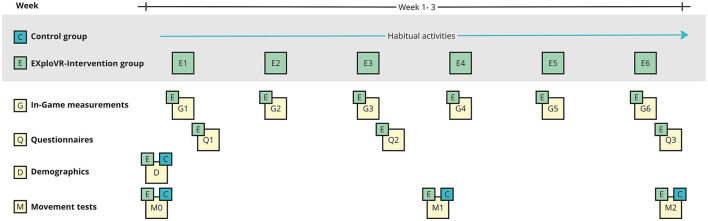
Study procedure for EXploVR.

At the study's baseline, both groups completed a demographic questionnaire and participated in movement assessments at three intervals. For the intervention group, movement assessments were conducted at three distinct intervals: at baseline (before the first session), mid-intervention (before the fourth session), and post-intervention (after the sixth session). The control group was assessed at the same intervals. Additionally, in-game measurements were collected during each training session for the intervention group. Questionnaires assessing experiences were administered after the first and third sessions and at the post-intervention assessment, specifically for the intervention group (see Figure 3). Adherence was tracked through an attendance log recorded at each session.

### 2.4 Measurements

#### 2.4.1 Demographics

Basic demographic information, including *age, sex*, and *weight*, was collected. *Educational background* was quantified as years of formal education based on typical German educational pathways: no school degree (8 years), basic school degree (Volks-/Hauptschulabschluss, 9 years), intermediate school degree (Realschul-/Fachschulabschluss, 10 years), higher secondary degree (Abitur/Fachabitur, 13 years), and university degree (Diplom, 17 years). Furthermore, participants reported their current *weekly training hours*. Participants were also asked about their *digital experience*, with all responses recorded on a 5-point Likert scale: frequency of digital media use (never, about once a month, several times a month, several times a week, multiple times daily), frequency of digital game use (never, about once a year, about once a month, about once a week, and daily), and VR experience (no experience, beginner—tried a few times, moderate experience, experienced, and expert).

#### 2.4.2 Digital Trail-Making Test

The *digital Trail-Making Test* (dTMT) is an Android application adapted from the paper-pencil *Trail Making Test* (TMT) by Reitan (1958), conducted on a tablet (Park and Schott, 2022). The dTMT assesses cognitive processing speed, executive function, attentional components, and fine motor performance. It consists of three parts: A, B, and M. Parts A and B follow the traditional TMT structure (Reitan, 1958; Salthouse, 2011), where in part A, participants connect numbered circles from 1 to 25 in ascending order as quickly as possible. In part B, they alternate between numbers (1 to 13) and letters (A to L) in ascending order at maximum speed. The third part, designated as M, involves a motor speed-tracking task. In this task, participants are instructed to trace empty circles connected by lines as rapidly as possible (Schott et al., 2016). The duration of this task, measured in seconds, is recorded for analysis.

#### 2.4.3 Outcome measures

##### 2.4.3.1 Effectiveness

Movement variables were measured using a portable inertial sensor system (Opal Sensors, APDM Mobility Lab, APDM Inc., Portland, OR, USA). Five inertial sensors were positioned on the lower back (spine at the lumbosacral junction), left and right feet (dorsum), and left and right wrists, secured with Velcro belts and straps. Data were collected at a sampling rate of 128 Hz, synchronized, and wirelessly transmitted to a laptop computer. This system provides a reliable and valid method for assessing gait and balance performance in clinical settings (Horak et al., 2015). Several APDM Mobility Lab tests have been performed using this setup: Instrumented Walk Tests, Instrumented Postural Sway, and Instrumented 5-times Sit-to-Stand Test.

The *Instrumented Walk Tests* assessed *gait speed* (m/s) under single-task and dual-task conditions, using normal and tandem walking trials. A 7-meter walking corridor with 180-degree turns at each end was used, with each trial lasting 60 seconds. In addition, a single-task cognition measurement was performed by *counting backwards in steps of three from a given number* for 60 seconds. The total *number of correct responses* and *errors* were recorded. This cognitive task was performed simultaneously with the walking task in the dual-task condition. Four different three-digit numbers were assigned randomly, with each participant encountering each number equally often.

The *Instrumented Postural Sway Test* assesses static postural control using a lumbar sensor in three stances: parallel, tandem, and semi-tandem. Participants are instructed to maintain balance for 30 seconds in each stance, with their hands at their sides. This test allows for the measurement of *RMS sway* (m/s^2^), which reflects the root mean square of the sway angle in the coronal and sagittal planes, *sway area* (m^2^/s^4^), defined as the area of an ellipse covering 95 % of the sway angle in these planes, and *path length* (m/s^2^), which captures the total length of the sway path in the transverse plane.

The *Instrumented Five Times Sit-to-Stand Test* (5xSTS) evaluates lower limb strength and transitional movement ability. Participants were instructed to stand and sit five times as quickly as possible with their arms crossed over their chest while seated with their back against an armless chair. Overall *completion time, sit-to-stand duration*, and *stand-to-sit duration* in seconds were recorded for analysis.

The exergame collects *time to completion* (s) for each station as an *in-game measurement* during training.

Additional *custom questions* were posed concerning the perceived mental and physical exertion within the exergame. The responses to these inquiries were meticulously documented using 5-point Likert scales.

##### 2.4.3.2 Enjoyment

The *Flow Short Scale* (FKS) (Rheinberg et al., 2019) is a questionnaire designed to measure the flow experience during an activity, assessing the extent to which individuals become fully engaged. In this study, the German version of the FKS was used. This 13-item instrument evaluates three factors using a 7-point Likert scale from “does not apply” to “fully applies”: *Smooth automated progression*, which reflects the sense that an activity feels effortless and flows almost automatically; *Absorption*, capturing complete immersion and deep involvement in the activity; and *concern*, measuring the absence of worries or self-doubt during the task. The combination of smooth automated progression and absorption determines *flow experience*. The Flow Experience scale demonstrated high internal consistency with a Cronbach's α of 0.88. The Smooth Automated Progression and Absorption sub-scales both showed excellent internal consistency with a Cronbach's α of 0.91. Cronbach's α for Concern was 0.62.

The PACES-S is a modified German *short version of the Physical Activity Enjoyment Scale*, designed to measure the enjoyment of physical activity (Chen C. et al., 2021; Fritsch et al., 2022). This version includes four items rated on a five-point Likert scale, ranging from “strongly disagree” to “strongly agree,” demonstrating excellent internal consistency (α = 0.93). Our approach has adapted the questions to account for cognitive (Cronbach's α = 0.95) and emotional challenges (Cronbach's α = 0.97).

The EEG-G is the validated German version of the interdisciplinary *Exergame Enjoyment Questionnaire*, specifically designed to assess enjoyment in playing exergames and intended for use immediately after gameplay. The questionnaire consists of 20 items addressing various aspects of the experience: immersion, intrinsically rewarding activity, control, and physical exercise. Responses are given on a 5-point Likert scale ranging from “strongly disagree,” “somewhat disagree,” “neutral,” “somewhat agree,” to “strongly agree” (Fitzgerald et al., 2020; Manser et al., 2023). The scale demonstrated good internal consistency (α = 0.82).

##### 2.4.3.3 Meaningfulness

The ABC-6 is a short, validated German version of the *Activities-specific Balance Confidence* questionnaire, comprising 6 items (Schott, 2014). This tool measures individuals' self-efficacy in their ability to perform daily activities without losing balance or risking falls. The questionnaire addresses everyday scenarios where balance is essential. In the present study, the test was expanded by adding two items to assess confidence in more advanced activities of daily living, specifically walking and hiking outdoors (ABC-8). Responses are recorded on a scale from 0% (no confidence) to 100% (complete confidence) for each activity. Cronbach's Alpha for ABC-6 and ABC-8 was α = 0.88, indicating good internal consistency.

Additionally, *custom questions* were posed to ascertain subjective perceptions and experiences within the exergame itself, including the extent to which participants felt safe during training and their fear of falling. To assess the meaningfulness of the exergame for daily life, questions included the perceived effectiveness of the exergame training to prepare for recreational activities like walking or hiking, how likely they are to integrate this training into their daily routine to feel safe in everyday life, and the importance of emotional challenges in daily life. All responses were recorded on a 5-point Likert scale.

### 2.5 Statistical analyses

#### 2.5.1 Data management

The in-game measurements for time to completion within the intervention group were analyzed by selecting each participant's first, middle, and last trials within each station. Completion times for each station were normalized using the mean completion times of intervention 1 (G1) as baseline measures. The 36 stations were divided into six thematic station sets, which were defined in the exergame design as related task types. Questionnaire responses were analyzed by calculating means and standard deviations or summing item scores, depending on the questionnaire design. Where applicable, subgroups were distinguished. Internal consistency was assessed using Cronbach's α. Movement data was analyzed using Mobility Lab software and raw data, with any data deemed to be corrupted excluded.

#### 2.5.2 Data analysis

Statistical analyses were conducted using Python. QQ plots and Levene tests were used to assess data distribution and homogeneity. Box-Cox transformation was employed to stabilize variance when the assumption of variances' homogeneity was violated. Descriptive analysis was performed, presenting means and standard deviations. A mixed-design analysis of variance (Mixed ANOVA) was conducted to assess differences across three time points (treated as the within-subject factor) and between the intervention and control group (treated as the between-subject factor). Separate analyses were performed exclusively for the intervention group, using a repeated-measures ANOVA with the three time points as the within-subject factor. Additionally, linear regression analysis using the Ordinary Least Squares (OLS) method was employed to investigate whether sex influenced study outcomes, including potential interactions with the factors of time points and group, where applicable. The Greenhouse-Geisser correction was applied if sphericity was violated. *Post hoc* analysis was performed using pairwise t-tests to identify specific differences for significant main effects and interactions. It is important to note that the intention-to-treat analysis was not considered in this analysis due to the explicit documentation of the reasons for dropout (Moher et al., 2012).

## 3 Results

### 3.1 Participant recruitment and retention

**Figure 4:**
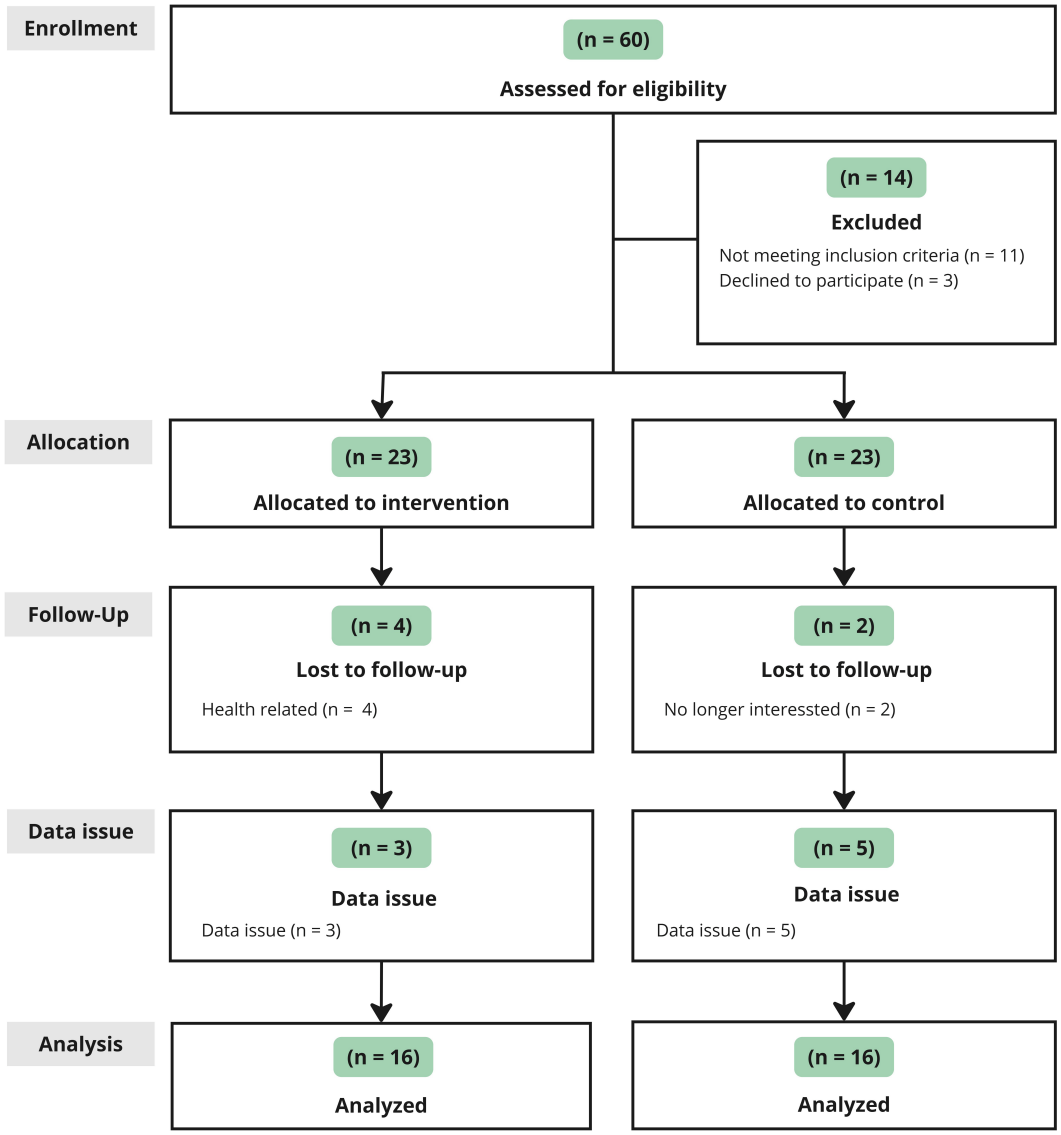
Flow chart of study.

A total of 60 participants were initially considered for inclusion in the study (see Figure 4). However, 14 participants were excluded from the study due to noncompliance with the inclusion criteria (*n* = 11) or declining participation (*n* = 3). The remaining 46 participants met the study's eligibility criteria and were thus included. These participants were then assigned to either the intervention group (*n* = 23) or the control group (*n* = 23).

A total of 32 participants completed the study, including 16 from the intervention group and 16 from the control group. Six participants were lost to follow-up during the study (intervention group: *n* = 4; control group: *n* = 2), resulting in an overall dropout rate of only 13%. In the intervention group, four participants were excluded due to health issues unrelated to the intervention, primarily linked to the flu season. Two participants in the control group withdrew due to a lack of interest. Additionally, data issues resulting from a software error in recording movement data led to the exclusion of three participants from the intervention group and five from the control group. Baseline demographic characteristics, lifestyle factors, digital experience, and cognitive parameters were well-balanced between the intervention and control group, as summarized in Table 2. It is worth mentioning that sex has no significant influence on outcome parameters within time points and between groups.

**Table 2:** Baseline characteristics of all participants (*n* = 32).

**Variable**	**Intervention group (*n* = 16)**	**Control group (*n* = 16)**	**p**
Age (years)	70.00 ± 3.33	68.38 ± 5.54	0.324
Sex, female/male (n)	9/7	7/9	0.724
Weight (kg)	72.19 ± 12.84	75.81 ± 11.23	0.402
Education (years)	13.81 ± 3.45	13.25 ± 2.96	0.624
Physical activity (min/week)	232.34 ± 222.39	145.00 ± 114.89	0.173
Digital media use (1–5)	4.81 ± 0.54	4.88 ± 0.50	0.737
Digital game use (1–5)	2.00 ± 1.55	2.00 ± 1.67	< 1.000
VR experience (1–5)	1.69 ± 0.95	1.75 ± 0.77	0.839
dTMT (M-inf) (s)	37.69 ± 18.72	37.40 ± 29.37	0.974
dTMT (A-inf) (s)	39.26 ± 9.58	42.27 ± 21.19	0.610
dTMT (B-inf) (s)	94.25 ± 44.35	68.27 ± 41.33	0.097

Values are means and standard deviations unless stated otherwise.

### 3.2 Effectiveness

The outcome measures, including mean values, standard deviations, and statistical results for single-task and dual-task conditions as well as secondary outcomes are presented in Table 3.

**Table 3:** Comparison of outcome measures between intervention and control groups for single-task and dual-task gait and cognition, and secondary outcomes.

**Task**	**Intervention group**			**Control group**			**F**	**p**	** $\eta_{p}^{2}$ **
	**M0** ^a^	**M1** ^a^	**M2** ^a^	**M0** ^a^	**M1** ^a^	**M2** ^a^			
**Single-task gait and cognition**									
Walking - Gait speed (m/s)	1.12 ± 0.17	1.15 ± 0.24	1.15 ± 0.22	1.38 ± 0.26	1.28 ± 0.27	1.25 ± 0.23	4.22	0.019^**^	0.12
Tandem - Gait speed (m/s)	0.56 ± 0.10	0.57 ± 0.11	0.64 ± 0.17	0.62 ± 0.06	0.61 ± 0.07	0.59 ± 0.07	3.66	0.032^**^	0.11
Counting bw (*n*)^b^	24.75 ± 10.08	27.31 ± 9.12	28.06 ± 8.03	24.19 ± 7.93	27.94 ± 8.14	31.12 ± 9.60	1.69	0.193	0.05
**Dual-task gait and cognition**			
Walking - Gait speed (m/s)	0.70 ± 0.16	0.70 ± 0.12	0.74 ± 0.16	0.78 ± 0.20	0.77 ± 0.17	0.80 ± 0.15	0.08	0.927	< 0.01
Tandem - Gait speed (m/s)	0.60 ± 0.15	0.59 ± 0.12	0.65 ± 0.17	0.71 ± 0.18	0.65 ± 0.16	0.60 ± 0.09	4.07	0.022^**^	0.12
Counting bw while walking (*n*)^b^	23.50 ± 7.36	24.75 ± 8.61	26.19 ± 10.22	23.44 ± 5.40	26.50 ± 7.26	28.25 ± 7.10	0.83	0.440	0.03
Counting bw while tandem (*n*)^b^	23.44 ± 8.28	24.81 ± 10.13	27.31 ± 7.60	24.31 ± 6.51	26.81 ± 8.34	29.44 ± 7.50	0.33	0.722	0.01
**5x sit-to-stand**			
Duration (s)	17.21 ± 3.37	15.72 ± 3.38	15.59 ± 3.45	13.88 ± 4.00	14.62 ± 3.87	14.89 ± 3.38	4.02	0.023^**^	0.12
Sit to stand - duration (s)	1.22 ± 0.32	1.16 ± 0.22	1.13 ± 0.23	1.04 ± 0.23	1.07 ± 0.25	1.10 ± 0.19	1.91	0.157	0.06
Stand to sit - duration (s)	1.15 ± 0.43	0.93 ± 0.22	0.93 ± 0.23	0.86 ± 0.25	0.88 ± 0.25	0.86 ± 0.24	2.37	0.103	0.08
**Postural sway parallel**			
RMS sway (m s^2^)	0.11 ± 0.05	0.10 ± 0.06	0.09 ± 0.03	0.09 ± 0.03	0.11 ± 0.05	0.09 ± 0.02	0.63	0.539	0.02
Sway area (m^2^ s^4^)	0.10 ± 0.07	0.09 ± 0.08	0.06 ± 0.05	0.07 ± 0.05	0.08 ± 0.05	0.07 ± 0.03	0.96	0.389	0.03
Path length (m s^2^)	9.42 ± 5.41	8.57 ± 3.72	7.04 ± 2.44	8.97 ± 2.96	9.22 ± 2.77	8.60 ± 1.85	0.73	0.487	0.02
**Postural sway semi-tandem**			
RMS sway (m s^2^)	0.15 ± 0.15	0.15 ± 0.07	0.12 ± 0.04	0.10 ± 0.03	0.11 ± 0.03	0.11 ± 0.07	0.74	0.480	0.02
Sway area (m^2^ s^4^)	0.16 ± 0.17	0.15 ± 0.10	0.13 ± 0.09	0.08 ± 0.04	0.09 ± 0.05	0.11 ± 0.12	0.74	0.482	0.02
Path length (m s^2^)	19.42 ± 15.20	17.77 ± 5.00	15.20 ± 5.16	13.01 ± 4.33	12.79 ± 4.20	13.42 ± 5.50	0.85	0.433	0.03
**Postural sway tandem**			
RMS sway (m s^2^)	0.25 ± 0.19	0.18 ± 0.09	0.22 ± 0.14	0.14 ± 0.10	0.15 ± 0.07	0.15 ± 0.12	1.40	0.255	0.04
Sway area (m^2^ s^4^)	0.34 ± 0.37	0.28 ± 0.19	0.28 ± 0.19	0.14 ± 0.12	0.17 ± 0.10	0.13 ± 0.09	0.93	0.400	0.03
Path length (m s^2^)	37.51 ± 20.41	35.03 ± 15.30	36.59 ± 17.09	26.23 ± 13.16	30.05 ± 13.42	28.81 ± 27.06	0.43	0.651	0.01

*F*-values, *p*-values, and partial effect sizes ($\eta_{p}^{2}$) represent group × time interaction effects.

^a^Mean and standard deviation.

^b^Counting backwards in steps of three from three-digit numbers.

^**^P < 0.05.

$\eta_{p}^{2}$ = Partial Effect Size.

#### 3.2.1 Instrumented tests

Under single-task conditions, significant group × time interactions were observed for normal walking gait speed (*p* = 0.019, $\eta_{p}^{2}=0.12$) and tandem gait speed (*p* = 0.019, $\eta_{p}^{2}=0.12$), indicating moderate effect sizes. At baseline, significant group differences were already present (walking *p* = 0.003, tandem gait *p* = 0.047). Over time, gait speed decreased in the control group. Under dual-task conditions, a significant group × time interaction was found for tandem gait speed ($p=0.022,\eta_{p}^{2}=0.12$), reflecting a moderate effect in favor of the intervention group. The *post hoc* comparisons suggest that these differences are not concentrated between specific trials for each group. No interaction was observed for walking gait speed ($p=0.923,\eta_{p}^{2}<0.01$), although the intervention group showed a slight, non-significant improvement. Cognitive performance improved slightly in both groups under single- and dual-task conditions but did not show statistical significance (*p*>0.1).

The 5xSTS task showed a significant improvement for total duration in the intervention group ($p=0.023,\eta_{p}^{2}=0.12$), indicating a moderate effect. At baseline, the total duration differed significantly between groups (*p* = 0.016). *Post hoc* comparisons within the intervention group revealed marginal improvement from trial 1 to trial 3 before correction ($p=0.076,\eta_{p}^{2}=0.46$), which did not remain significant after Bonferroni correction (*p* = 0.228). Transition times (sit-to-stand and stand-to-sit) showed no statistical significance.

Postural sway measures did not differ significantly between groups (*p*>0.1). However, small trends toward reduced sway area and path length were observed in the intervention group, particularly in parallel and semi-tandem stances.

#### 3.2.2 In-game measurements

**Figure 5:**
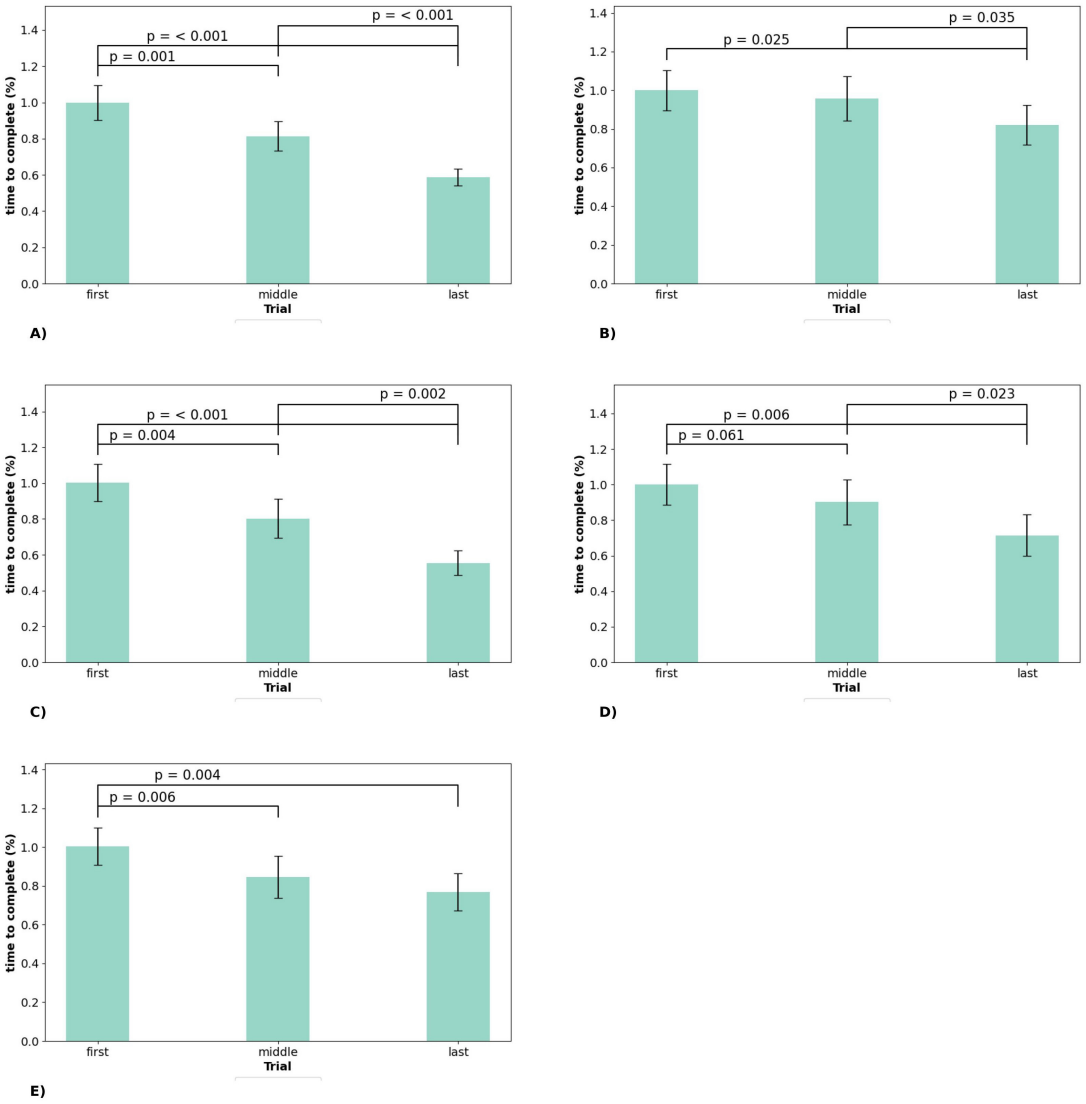
Significant outcomes for in-game measurements from *post hoc* analysis via pairwise testing. **(A)** Platform Navigator. **(B)** Rotor Walk. **(C)** Pattern Trail. **(D)** Pathfinder's Choice. **(E)** Narrow Twist Path.

Outcomes of normalized, time-dependent in-game measurements are presented in Table 4 and *post hoc* analysis with pairwise testing in Figure 5. In-Game completion time improved significantly across most Level 1 and Level 2 station sets. In Level 1, Platform Navigator (*p* < 0.001, $n_{g}^{2}=0.24$) and Pattern Trail (*p* < 0.001, $n_{g}^{2}=0.20$) demonstrated large effect sizes, while Rotor Walk showed a moderate effect (*p* < 0.012, $n_{g}^{2}=0.03$). In Level 2, Pathfinder's Choice (*p* < 0.002, $n_{g}^{2}=0.06$) and Narrow Twist Path (*p* < 0.003, $n_{g}^{2}=0.06$) showed moderate reductions in the percentage of completion times, while no significant changes were observed for Balancing Act (*p* = 0.481).

**Table 4:** Normalized mean values for the time to complete a station of the exergames, summarized by station sets.

**Station set**	**First trial^a^**	**Middle trial^a^**	**Last trial^a^**	**F**	**p**	** $\eta_{g}^{2}$ **
**Level 1**						
Platform Navigator - Time to complete (%)	1.00 ± 0.38	0.81 ± 0.33	0.59 ± 0.19	30.00	< 0.001^***^	0.24
Rotor Walk - Time to complete (%)	1.00 ± 0.42	0.96 ± 0.46	0.82 ± 0.41	5.14	0.012^**^	0.03
Pattern Trail - Time to complete (%)	1.00 ± 0.41	0.80 ± 0.44	0.55 ± 0.28	22.75	< 0.001^***^	0.20
**Level 2**						
Pathfinder's Choice - Time to complete (%)	1.00 ± 0.46	0.90 ± 0.51	0.71 ± 0.47	7.93	0.002^***^	0.06
Balancing Act - Time to complete (%)	1.00 ± 0.56	0.96 ± 0.52	0.87 ± 0.55	0.75	0.481	0.01
Narrow Twist Path - Time to complete (%)	1.00 ± 0.39	0.85 ± 0.43	0.77 ± 0.38	7.33	0.003^***^	0.06

^a^Mean and standard deviation.

**P < 0.05, ***P < 0.01.

$\eta_{g}^{2}$ = General Effect Size.

*Post hoc* analyses revealed that for Platform Navigator, completion times improved highly significantly between all trial comparisons (first vs. middle: *g* = 0.51, middle vs. last: *g* = 0.83, first vs. last: *g* = 1.34; all *p* ≤ 0.001), indicating moderate to very large effect sizes. Pattern Trail also showed highly significant improvements (first vs. middle: *g* = 0.46, middle vs. last: *g* = 0.66, first vs. last: *g* = 1.24; all *p* ≤ 0.001). Effect sizes for Pattern Trail ranged from small to very large. For Rotor Walk, significant differences were observed between the first and the last trial, as well as between the middle and the last trial (*p* < 0.05), with small effect sizes (*g* < 0.50). Pathfinder's Choice demonstrated marginal improvements between the first and middle trials (*p* = 0.061, *g* = 0.20), while significant improvements occurred between middle and last (*p* = 0.023, *g* = 0.34) and the first and last trials (*p* = 0.006, *g* = 0.60). Effect sizes ranged from small to moderate. Narrow Twist Path showed significant reductions between the first and middle trials (*p* < 0.01, *g* = 0.38) and the first and last trials (*p* < 0.01, *g* = 0.59), with small to moderate effect sizes.

#### 3.2.3 Custom questions

Table 5 shows that the perceived mental effort was initially rated as moderate (2.56 ± 0.89), while the perceived physical effort was low in Trial 1 (Q1) (2.06 ± 0.85). Both measures remained stable, with no significant changes observed (*p*>0.1).

**Table 5:** Comparison of questionnaires between the trials of the intervention group for flow experience, PACES, ABC, and custom questions.

**Variable**	**Q1^a^**	**Q2^a^**	**Q3^a^**	**F**	**p**	** $\eta_{g}^{2}$ **
**Custom questions effectiveness** ^††^						
Perceived mental effort	2.56 ± 0.89	2.67 ± 0.62	2.67 ± 0.72	0.32	0.731	0.01
Perceived physical effort	2.06 ± 0.85	2.33 ± 0.98	2.20 ± 1.08	0.57	0.575	0.01
**Flow experience (FKS)** ^†^						
Flow experience	5.18 ± 1.04	5.29 ± 1.01	5.68 ± 0.74	2.69	0.085*	0.04
Smooth automated progression	4.88 ± 1.29	5.07 ± 1.11	5.57 ± 0.99	3.84	0.034^**^	0.06
Absorption	5.64 ± 0.94	5.62 ± 0.93	5.84 ± 0.85	0.28	0.758	0.01
Concern	2.81 ± 1.76	2.73 ± 1.96	3.19 ± 2.24	0.41	0.671	0.01
**PACES** ^‡^						
Enjoyment of physical challenges	16.19 ± 4.31	14.80 ± 4.11	16.25 ± 3.97	3.03	0.064*	0.02
Enjoyment of cognitive challenges	16.19 ± 3.78	16.27 ± 3.03	16.88 ± 3.72	0.12	0.886	0.00
Enjoyment of emotional challenges	15.31 ± 4.36	15.33 ± 4.72	14.44 ± 5.30	0.87	0.430	0.01
**EEQ-G** ^¶¶^						
EEQ-Enjoyment	78.69 ± 8.94	78.00 ± 8.74	81.19 ± 9.09	1.31	0.286	0.02
**ABC** ^¶^						
ABC-6	75.10 ± 16.76	77.56 ± 15.04	78.81 ± 18.31	2.61	0.094^*^	0.02
ABC-8	62.93 ± 15.39	67.50 ± 16.92	69.24 ± 20.73	5.00	0.026^**^	0.09
**Custom questions meaningfulness** ^††^						
Perceived safety during training	3.56 ± 1.21	3.20 ± 1.15	3.27 ± 1.39	1.39	0.268	0.03
Fear of falling during training	2.12 ± 0.81	2.33 ± 0.82	1.93 ± 0.88	5.89	< 0.01^***^	0.09
Perceived effectiveness of training for preparation	2.94 ± 1.12	2.73 ± 1.39	3.07 ± 0.96	0.10	0.905	0.00
Integration into everyday life for safety	2.69 ± 1.01	2.27 ± 0.80	3.13 ± 0.99	3.60	0.042^**^	0.12
Importance of emotional challenges in daily life	2.56 ± 0.96	2.20 ± 0.94	3.00 ± 1.00	2.01	0.154	0.09

^a^Mean and standard deviation.

^*^P < 0.1, ^**^P < 0.05, ^***^P < 0.01.

^†^Scale range 1–7 (best score 7).

^‡^Scale range 1–5 (best score 20).

^¶¶^Scale range 20–100 (best score 100).

^††^Scale range 1–5 (best score 5).

^¶^Scale range 0–100 (best score 100).

$\eta_{g}^{2}$ = General effect size.

### 3.3 Enjoyment

#### 3.3.1 Flow short scale

Flow experience was initially high (5.18 ± 1.04 out of 7) in questionnaire trial 1 (Q1) and it increased marginally significant over time ($p=0.085,n_{g}^{2}=0.04$). The smooth automated progression subscale was rated as moderate (4.88 ± 1.29) in Q1 and exhibited significant improvement throughout exergame use, with a medium effect size ($p=0.034,n_{g}^{2}=0.06$). The score for absorption in Q1 was high (5.64 ± 0.94) and remained consistently high and stable over time (*p* = 0.758). In addition, the score for Concern was low (2.84 ± 1.71) in Q1 and remained consistently low over time (*p* = 0.671).

#### 3.3.2 Short version of the physical activity enjoyment scale and adaptations

The PACES was rated highly in Q1, with a score of 16.19 ± 4.31 out of 20. Over time, the score showed a marginally significant change (*p* = 0.064). Similarly, the enjoyment of cognitive and emotional challenges received high scores (cognitive: 16.19 ± 3.78, emotional: 15.31 ± 4.36) and remained stable using the exergame (*p*>0.1).

#### 3.3.3 Exergame enjoyment questionnaire

The score for exergame enjoyment was high in Q1, with a mean of 78.69 ± 8.94 out of 100, and remained consistently high over time (*p* = 0.286).

### 3.4 Meaningfulness

#### 3.4.1 Short versions of the activities specific balance confidence scale

Balance confidence for basic activities, measured by ABC-6, was initially high (75.10 ± 16.76 out of 100) and showed a marginally significantly improvement over time (*p* = 0.094). Confidence in advanced activities, assessed by the ABC-8, started at a moderate level (62.93 ± 15.39) and improved significantly throughout the intervention (*p* = 0.026, $n_{g}^{2}=0.09$), reaching a high confidence level (69.24 ± 20.73).

#### 3.4.2 Custom questions

Perceived safety during training was rated as high in Q1, with a score of 3.56 ± 1.21 out of 5, and remained stable over time (*p* = 0.268). Fear of falling during training was initially low (2.12 ± 0.81) and decreased significantly throughout the study (*p* < 0.01). Integration into everyday life for safety received an initial moderate rating (2.69 ± 1.01) and improved significantly over time (*p* = 0.042, $\eta_{g}^{2}=0.12$). Moderate Q1 ratings were also observed for the perceived effectiveness of training for preparation (2.94 ± 1.12) and the importance of emotional challenges in daily life (2.56 ± 0.96), both of which remained stable over study time (*p*>0.1).

## 4 Discussion

This exploratory study investigated the interdisciplinary VR gait-based exergame EXploVR, which was developed to prevent falls in community-dwelling healthy older adults over a 3-week intervention period. The results indicated that EXploVR has the potential to deliver measurable benefits in three key areas: effectiveness, enjoyment, and meaningfulness (EEM).

### 4.1 Effectiveness

EXploVR led to improvements in gait performance, lower limb strength, and the ability to manage dual-task situations, which may contribute positively to fall prevention in healthy older adults.

EXploVR demonstrated promising effects on gait performance, particularly under challenging conditions. Improvements in dual-task tandem gait speed, with moderate effect size, can be regarded as substantial changes, aligning with the documented low to moderate effects of gait speed in older adults (Perera et al., 2006). Such changes are associated with a reduced risk of falling. Furthermore, the observed improvements are consistent with previous research showing increased dual-task gait speed in healthy older adults following semi-immersive VR training (Zukowski et al., 2022). Significant effects emerged for single-task gait speed; however, *post hoc* comparisons did not reveal statistically significant within-group changes, but indicated encouraging trends toward early gait adaptability. These findings highlight the particular relevance of tandem gait assessments (Park and Schott, 2025). As the human body undergoes the natural aging process, postural control changes become more evident during tandem gait than during normal walking or quiet standing. This is because tandem gait requires a reduced support surface, which in turn challenges the postural control system (Fu et al., 2021). Consequently, tandem walking provides a sensitive means to assess aspects of dynamic balance and mobility that may not be fully captured by conventional walking and standing tests. This approach can potentially reveal additional insights into the effects of aging and pathologies that may not be evident in more traditional testing methods. In this regard, it is imperative to integrate these tests with additional sensor data as fall-relevant parameters, a practice that has been adopted in this study. In-game completion times improved significantly across most station sets, including Platform Navigator, Rotor Walk, Pattern Trail, Pathfinder's Choice, and Narrow Twist Path, with significant differences between the first and last trials in *post hoc* analysis. Furthermore, station sets targeting dual-task gait variations and body rotation (Rotor Walk, Pattern Trail, and Pathfinder's Choice) significantly improved from the middle to the last trials. These findings suggest that the observed changes relate not merely to initial familiarization effects but also to learning processes or training adaptations. This could be attributed to ExploVR's feedback methods, which provide immediate responses to user actions, as well as a constant progression in difficulty, variable practice conditions, and task-specific training, all of which support motor learning through targeted learning experiences (Demers et al., 2021). Further support was observed in the station sets Platform Navigator and Narrow Twist Path, which emphasize dynamic postural control with a focus on mobility (e.g., weight shifting and stepping in different directions). Significant *post hoc* improvements were noted in both station sets, with Platform Navigator demonstrating particularly strong effects: a large effect size between the middle and last trial, and a very large effect size between the first and last trial. The results of the gait-related parameters aligned with our expectations, as this pilot study involved active and healthy older adults. Their baseline single-task walking gait speed exceeded the threshold values of 1.0 m/s (Cesari et al., 2005) and 1.02 m/s (Callisaya et al., 2011), which are known predictors of adverse outcomes including falls in older adults. Additionally, participants reported high weekly exercise levels, reflecting high physical fitness (Fisher et al., 2018), corresponded with their feedback that the exergame tasks were perceived as physically undemanding yet cognitively moderately challenging. This pilot study's relatively brief 3-week intervention period might have also limited measurable training effects (Fang et al., 2020; Fernandes et al., 2022). A recent VR study (Ghous et al., 2024) demonstrated significant improvements in the timed-up-and-go test and the dynamic gait index following an eight-week treatment, which consisted of 24 sessions of 30 minutes each, administered three days per week for the first four weeks. The treatment period was then extended for another four weeks, during which time periods gradually increased from 30 to 40 min.

Lower limb strength improvements were indicated by a 9.4% increase in performance from M0 to M2 in the 5xSTS test. Although *post-hoc* analysis revealed a small to moderate significant effect, this did not remain significant after Bonferroni correction. Additionally, highly significant improvements in in-game completion time were observed in the station sets focusing on lower limb engagement, particularly Platform Navigator and Narrow Twist Path. The modest enhancements in muscle strength may be attributable to processing information from the visual and somatosensory systems within the VR environment that may have enhanced challenges through higher cognitive demands (Sadeghi et al., 2021).

Observations of dual-task tandem gait speed indicated enhancements in cognitive-motor integration under complex task conditions, suggesting improvements in training for dual-task situations. In contrast, no notable improvements were observed for dual-task walking performance. This discrepancy may be due to participants' well-trained status. The more challenging dual-task tandem walk likely allowed for greater improvement due to its higher cognitive and motor demands, unlike the more automated and less demanding dual-task walking (Strouwen et al., 2019). Supporting this explanation, the pure cognitive task of backward counting in steps of three from three-digit numbers showed no significant differences between single-task and dual-task conditions. Furthermore, learning effects in backward counting were observed in the intervention group and the control group. This may be attributed to a lack of transfer effects, as backward counting was not explicitly trained within the exergame.

No significant improvements in static postural control were observed, as slight trends in postural sway lacked statistical significance and relevant effect sizes. High variability hindered clear identification of intervention effects. A recent review on the benefits of VR training for balance and gait (Rodŕıguez-Almagro et al., 2024) also showed only partial positive effects on balance parameters in older adults, including improvements in static and dynamic balance, gait, and an increase in lower limb strength.

### 4.2 Enjoyment

The exergame maintained consistently high enjoyment levels throughout the intervention among healthy, community-dwelling older adults.

A pervasive challenge in exergames is the waning of user motivation over time (Chan et al., 2019). Thus, maintaining high enjoyment levels is essential for sustaining motivation. This is reflected in the questionnaires' responses, which consistently place enjoyment ratings in the upper third of the scales. The results show that enjoyment scores remain consistently high or even improve over the study period. Importantly, while older adults frequently exhibit initial reservations toward VR technology, as evidenced in the extant literature (Healy et al., 2022; Huygelier et al., 2019), this demographic's level of enjoyment was attained.

High ratings in enjoyment were observed in the interdisciplinary EEQ-G, which evaluates aspects of user experience, game design, and human movement science (Fitzgerald et al., 2020). These positive outcomes can be attributed to the development of ExploVR following the interdisciplinary CIEMER design model (Retz et al., 2024). Additionally, PACES results indicate that enjoyment derived from physical, cognitive, and emotional challenges is addressed equally. However, it is worth noting that only the physical questionnaire variation of PACES has been validated outside the scope of this study. While the cognitive and emotional variations use the same question structure, their validation remains confined to this specific context. The internal consistency was predominantly high, with notably elevated values observed in the emotional challenge scale, potentially signifying redundancy in the items.

In addition to consistently high ratings, overall flow experience and the smooth automated progression subscale increased significantly during the intervention, suggesting a strong sense of presence and offering powerful affordances for facilitating a flow state (Hassan et al., 2020).

### 4.3 Meaningfulness

The exergame demonstrated its potential to be meaningful during the training and to extend to real-life situations.

Participants reported a high sense of safety while engaging with the exergame, and a significant reduction in fear of falling was observed throughout the intervention despite already low initial levels. It can be hypothesized that this is due to the high initial levels of the sample. It is also important to acknowledge that the measurement was based on a single question, not a validated questionnaire. Confidence in advanced activities (ABC-8) was moderate initially and increased significantly over time. Notably, we extended the original short version of the German ABC-6 scale by two items as no suitable existing questionnaire was available. Despite the high Cronbach's alpha and consistency with the ABC-6, the validation of the scale remains constrained to the present study. Falls-associated self-efficacy (ABC-6) exhibited moderate to high levels at baseline, but its stability as an assessment of fall-associated self-efficacy led to only a slight improvement. These outcomes align with the current levels of training and self-assessed fitness (Schott, 2007, 2022). The exergame's capacity to positively influence integration into real-life activities is further substantiated by the responses to the user-defined question concerning integration into everyday life for safety purposes.

## 5 Strengths and limitations

A critical evaluation of the pilot study reveals several noteworthy strengths and limitations. A key strength is the innovative intervention, which integrates the objectives of EEM alongside natural gait techniques into a fully immersive VR exergame. The structured, concept- and procedure-based development process ensures reproducibility, transparency, and a clear rationale for the outcomes. Additional strengths include the rigorous methodology, with clearly defined inclusion and exclusion criteria, validated outcome measures, and specific custom questions. It is important to note that in-game measurements are not derived from validated tests, which may limit the generalizability of these findings. Additionally, the relatively brief intervention period of three weeks may have limited the ability to observe significant effects, and no long-term follow-up measurements were included. The quasi-randomized design may have introduced potential bias despite including two groups with comparable characteristics. Furthermore, the relatively small sample size and the participants' significantly higher exercise activity level than the average individual may limit the generalizability of the findings. Future studies should aim to recruit a larger and more diverse sample, particularly untrained yet healthy older adults. Movement-related evaluation methods should be carefully selected to better capture the effectiveness of the exergame in achieving its intended goals. Finally, qualitative research could complement the quantitative data by offering more profound insights into participant experiences and clarifying the design decisions behind the observed effects.

## 6 Conclusion

This study evaluated the effectiveness, enjoyment, and meaningfulness (EEM) of a fully immersive virtual reality natural gait-based exergame for fall prevention designed with the interdisciplinary CIEMER model. Conducted as a pilot study, it involved community-dwelling healthy older adults over a 3-week test period using a quasi-randomized design. The findings revealed improvements in effectiveness, particularly in single- and dual-task gait conditions, 5xSTS performance, and in-game measurements. Enjoyment levels, assessed through FKS, PACES-S (and variations), and EEQ-G, were consistently high, showing stability or increases over time. In the meaningfulness category, improvements were observed in balance confidence (ABC-6 and ABC-8 scales). Incorporating custom questions addressing perceived safety, fear of falling during training, integration into everyday life, and the importance of emotional challenges in daily life corroborated these findings, alongside the perceived effectiveness of fall prevention preparation. However, the EEM dimensions require further investigation through randomized controlled trials to confirm and expand upon these results. Future work could build on the EEM approach to develop training programs for healthy older adults and those outside community settings. Moreover, EXploVR may serve as a potential component of a digital platform for health-related applications.

## Data Availability

The original contributions presented in the study are included in the article/supplementary material, further inquiries can be directed to the corresponding author.
